# Transforming cardiology with AI: the eko CORE 500 digital stethoscope

**DOI:** 10.1007/s12471-025-01973-0

**Published:** 2025-07-31

**Authors:** Victor Umans

**Affiliations:** Department of Cardiology, Noordwest Ziekenhuisgroep Alkmaar, Alkmaar, The Netherlands

Dear Editor,

I was pleased to read the recent article ‘*Transforming Cardiology with AI* [1]*. *The article’s emphasis on the future of cardiology—particularly regarding the integration of digital technology into clinical practice—is both timely and inspiring.

As a clinician practicing in the field of cardiology, I would like to take this opportunity to highlight one additional clinical application that may have a profound impact on daily practice: the digital stethoscope’s ability to connect seamlessly via Bluetooth to modern hearing aid solutions.

In my own experience, integrating digital stethoscopes into clinical workflow has significantly improved diagnostic accuracy and patient care. For colleagues with hearing impairments, the ability to connect digital stethoscopes via Bluetooth to hearing aids is a game-changer. This integration not only enhances the clarity of auscultation but also ensures that healthcare professionals with hearing challenges are not at a disadvantage during routine cardiovascular examinations.

It is also worth noting that the CORE stethoscope is available in the Netherlands and has an appropriate CE mark.

Finally, the knowledge of a digital stethoscope and its connecting options is not known to hearing specialists of the main hearing aid solution companies.
